# 14.4 FOLLOW-UP TREATMENT FOR INDIVIDUALS WITH SERIOUS MENTAL ILLNESS WHO HAVE COMMITTED MAJOR CRIMES

**DOI:** 10.1093/schbul/sby014.057

**Published:** 2018-04-01

**Authors:** E Fuller Torrey

**Affiliations:** The Stanley Medical Research Institute

## Abstract

**Background:**

For individuals who have a psychiatric disorder and have committed a major crime, the rate of re-offending is twice as high in the US compared to nine other countries for which there is comparable data. For such individuals the average five-year rearrest rate is approximately 40% for those released from psychiatric hospitals and 60% for those released from jails or prisons. The use of treatment modalities such as extended conditional release, Forensic Assertive Community Treatment (FACT) teams, and Psychiatric Security Review Boards can reduce the rearrest rate from 40–60% to 10% or less.

**Methods:**

All 50 states were surveyed to assess how they were doing in providing follow-up treatment for such individuals.

**Results:**

Sixteen states were found to be making a moderate effort to provide follow-up treatment, and another 13 states are making a minimal effort. However, the other 21 states, 42% of the total, are making virtually no effort, lending to an unnecessarily high rate of re-offending.

**Discussion:**

Using proven treatment approaches the re-arrest rate of individuals with serious mental illness can be reduced from 40–60% to 10% or less.

